# Large traumatic vulvar hematoma managed with a simple incision: A case report

**DOI:** 10.1016/j.ijscr.2021.106080

**Published:** 2021-06-09

**Authors:** Brahmana Askandar Tjokroprawiro

**Affiliations:** Department of Obstetrics and Gynecology, Dr. Soetomo General Academic Hospital, Medical Faculty, Universitas Airlangga, Surabaya, Indonesia

**Keywords:** Vulvar hematoma, Vulvar trauma, Pelvic trauma, CT, computed tomography

## Abstract

**Introduction and importance:**

Non-obstetric vulvar hematoma is a rare condition. With only few cases reported, no standard consensus exists on the best management of vulvar hematomas. Herein, we report the successful management of a large trauma-induced vulvar hematoma through a simple incision under local anesthesia. Localized large vulvar hematomas can be treated with this simple procedure.

**Case presentation:**

A 38-year-old woman presented with vulvar pain following a fall. Investigations revealed a large painful hematoma in the right labium majus. Imaging revealed that the hematoma was localized and the pelvic structure was normal. We successfully treated the hematoma by making an incision in the right labium majus under local anesthesia to evacuate the clot and ligate the bleeding points. She was discharged in a good condition after two days.

**Discussion:**

Non-obstetrics vulvar hematoma is rare particularly the large hematoma. A blunt trauma may cause a large vulvar hematoma. There is no standard management of vulvar hematomas since the incidence is very low. The important step in managing vulvar hematoma is detecting the localization of the hematoma and evaluating other pelvic structures. Most of vulvar hematoma is isolated in the soft tissue around the labium majus and the vagina without further extension. After confirming that the large vulvar hematoma is localized and there are no injuries to the other pelvic structures, a simple incision and bleeding points ligation under local anesthesia can be performed to relieve the pain and prevent pressure necrosis.

**Conclusion:**

Simple incision under local anesthesia is effective for managing large vulvar hematomas and reduces the recovery time.

## Highlights

• Blunt trauma may cause a large vulvar hematoma
• Large vulvar hematoma is commonly localized and should be confirmed by imaging
• Incision for large localized vulvar hematoma will relieve the pain quickly and may prevent necrosis

## 1 Introduction

Vulvar hematoma caused by trauma is a rare clinical condition with an incidence of 0.8% among all gynecologic emergencies [1]. Only few cases of large vulvar hematomas were reported in literature [1,2]. There is no effective consensus on the management strategy for such hematomas, due to their rarity. Small hematomas can be managed conservatively, but there are some indications for surgical intervention. The surgical solution reduces morbidity and length of stay in the hospital [3]. While larger vulvar hematomas may give the impression of a severe condition, most are localized and may only require a simple procedure. Herein, we present a case of a large vulvar hematoma that was successfully treated with a simple incision under local anesthesia.

## 2 Case presentation

A 38-year-old woman living in a village in Indonesia reported to the hospital after a fall in which her inguinal area hit a steel floor. She was experiencing worsening pain in the vulvar area after the fall, but was still mobile and was not experiencing vaginal bleeding. In the emergency department, the doctor on duty evaluated the condition and noted that the hemodynamics were stable. Due to a lack of facilities and gynecologists, the patient was referred to a tertiary hospital in the second-largest city in Indonesia.

**Fig. 1:**
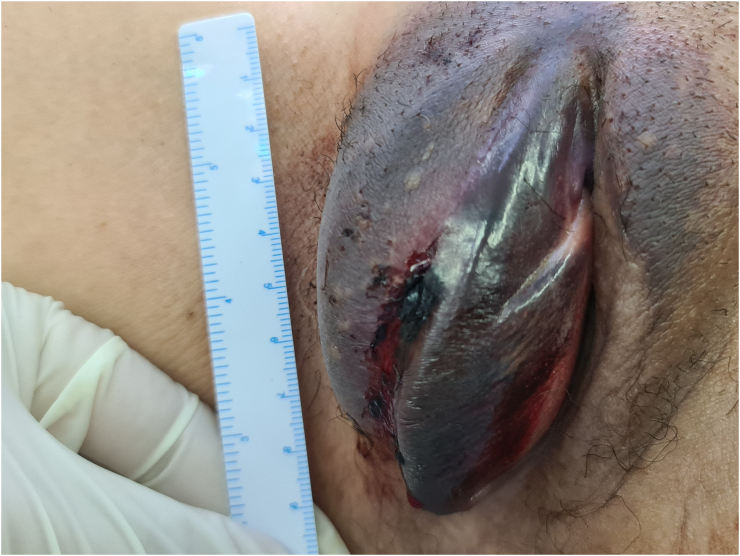
Large hematoma of the right labium majus.

**Fig. 2:**
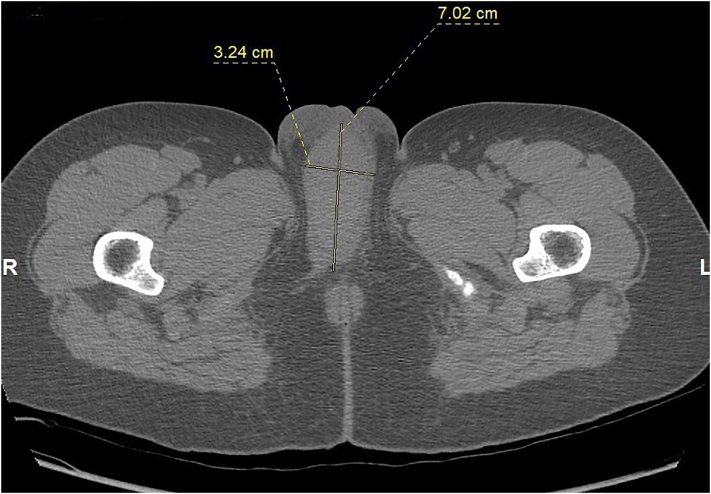
The axial computed tomography scan shows a large hematoma in the right labium majus with a diameter of 7.02 × 3.24 cm.

The patient arrived at the emergency department of the tertiary hospital 8 h after the incident. She had no history of allergies, drug use or any other medical disorder. Upon arrival, she was stable with a blood pressure of 131/74 mm Hg and a heart rate of 92 beats per minute. Evaluation of the external genitalia revealed a large hematoma and ecchymosis in the right labium majus (Fig. 1). No bleeding was observed, and the urethra and vagina appeared normal. The hematoma was growing and the patient was administered with 200 mg of ketoprofen rectally to manage the pain. Her hemoglobin level was 13.9 g/dL with a white blood cell count of 7540/μL. Non-contrast computed tomography (CT) was performed, which revealed a large localized hematoma in the right labium majus having a diameter of 7.02 × 3.24 cm (Fig. 2). Sagittal and coronal CT scans also revealed that the hematoma was localized in the vulvar area, and did not extend to the pelvis. The pelvic structures appeared normal.

**Fig. 3:**
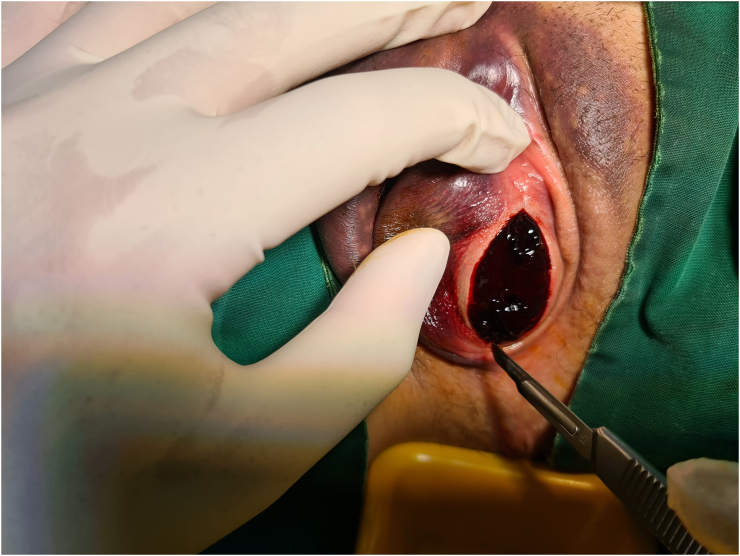
A 3-cm incision is made in the border between the mucosa and the skin in the right labium majus to evacuate the blood clot.

**Fig. 4:**
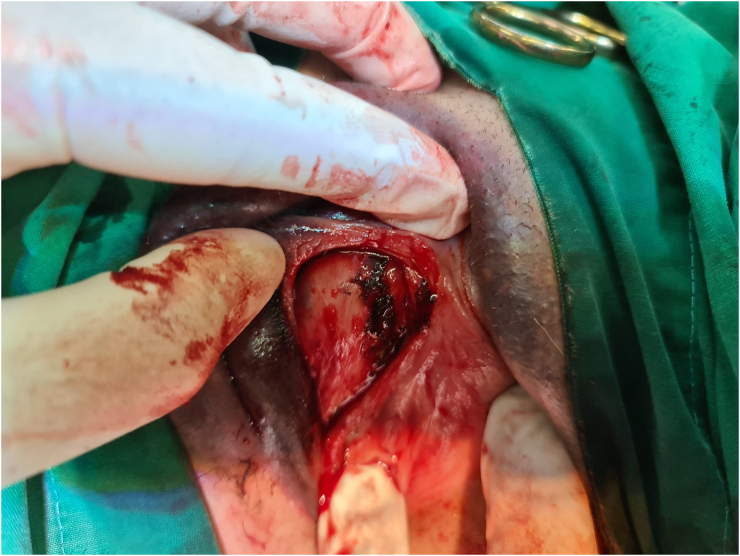
The cavity after blood clot evacuation. Bleeding points are observed.

**Fig. 5:**
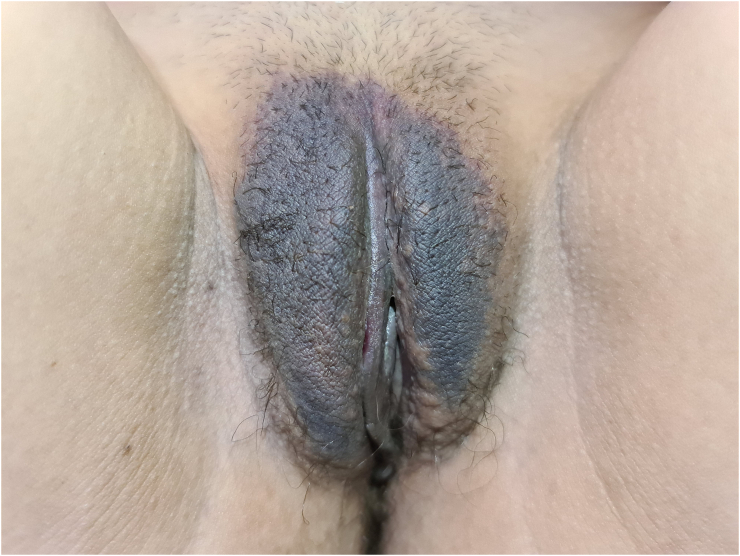
The condition of the vulva one day after incision.

Patient was consented and incision of the hematoma under local anesthesia was performed by an experienced gynecologic oncologist in the operating theatre. Lidocaine was injected in the right labium majus for local anesthesia, and a 3-cm incision was made at the border between the mucosa and the skin (Fig. 3). The blood clot was evacuated, and active bleeding points were observed around the clitoris and the perineum, which were stitched using rapid vicryl 2.0 (Johnson & Johnson, USA). The cavity was irrigated to clean the clot and evaluate whether all bleeding points had been managed (Fig. 4). The incision was closed with a running suture, and a 1-cm hole was left open for drainage. The patient was given oral amoxycillin/clavulanic acid for 7 days and hospitalized in the gynecologic ward for observation after the procedure. On day 1 after the procedure, the urethral catheter was removed and urination function was normal. The condition of the vulva one day after the incision is shown in Fig. 5. The patient was not experiencing pain and had complete mobility. No bleeding was observed from the incision site. The patient was discharged from the hospital on day two after the procedure in good condition. The outcome of the simple incision of the large vulvar hematoma under local anesthesia in this patient was good as expected. Patient was very happy, the large vulvar hematoma was managed successfully with a simple procedure. A two-week follow up revealed recovery. From the patient's perspective, large vulvar hematomas should be managed in a tertiary hospital with experienced specialists and complete facilities so that other women with the same condition can be managed safely. The patient described here provided written informed consent for this report. This case has been reported in line with the SCARE 2020 criteria [1].

## 3 Discussion

While many vulvar hematomas are related to obstetric complications, non-obstetric vulvar hematomas are an uncommon condition with an incidence of 0.8% among all gynecological emergencies [2]. Furthermore, 70% of all recorded vulvar hematomas occur on the right labium despite the lack of anatomical explanation [3]. As the incidence is very low, there is no standard clinical practice for the management of vulvar hematomas. Furthermore, it is still controversial whether vulvar hematomas should be managed conservatively or surgically.

The majority of vulvar hematomas are caused by injury to the branches of the pudendal artery, which is a branch of the internal iliac artery. Injury of the pudendal branch may occur in the anterior or posterior pelvic triangle. If the injury occurs in the anterior pelvic triangle, the extension of the hematoma is limited by the urogenital diaphragm and Colles' fascia (superficial perineal fascia), directing the hematoma to the loose subcutaneous tissue around the vulva. As such, vulvar hematoma is commonly isolated in the soft tissue around the labium majus and the vagina without further extension. In our patient, the hematoma was isolated in the right labium majus with bleeding points from the branches of the pudendal artery around the vulva. It is critical when identifying a vulvar hematoma to determine its extension, which typically requires imaging procedures such as ultrasound, CT, or magnetic resonance imaging. Furthermore, imaging techniques allow for the assessment of other organ damage or fractures. The imaging modality of choice for assessing trauma is CT [4] due to its high sensitivity and negative predictive value [5]. CT was found to be superior to pelvic X-ray for the detection of lumbar, pelvic, and femoral fractures [6], and is the primary imaging method for assessing the lower urinary tract for trauma [7]. CT scanning is thus, a critical step to exclude musculoskeletal, genitourinary, and pelvic vascular injuries before proceeding with treatment.

The best management strategy for vulvar hematomas is still widely debated. The choice of conservative or surgical management depends on the patient's condition and the size of the hematoma. Small hematomas without acute expansion can be managed conservatively by administering analgesics and antibiotics [2]; however, conservative management may require longer hospital stays, and is associated with an increased need of antibiotics and blood transfusion [8]. If the hematoma grows larger and the pain persists, surgical intervention is usually recommended [2]. Hematomas in the vulva that are larger than 4 cm in diameter or those accompanied by hemodynamic instability and/or persistent pain typically require surgical intervention [3]. In our case, the diameter of the hematoma was 6.5 cm, and the patient suffered from severe and worsening pain, indicating a need for surgical intervention. After confirming that the large vulvar hematoma was localized and there were no injuries to the other pelvic structures, we performed an incision in the right labium majus under local anesthesia to evacuate the clot and ligate the bleeding points. The goal of this incision was to relieve pain and prevent pressure necrosis. The large traumatic vulvar hematoma in previous report was managed under general anesthesia [3]. The other reported case was managed conservatively after spontaneous rupture of the large vulvar hematoma [2]. The patient in our case was managed with simple procedure. No standard consensus on the management of vulvar hematoma exists. This case revealed that despite the large size of traumatic vulvar hematomas, they can be treated through a simple incision under local anesthesia. Before deciding on simple incision, it should be confirmed that hematomas are localized, with no injury to the other pelvic structures.

While we were able to successfully identify the bleeding points in our patient, in situations where the bleeding points cannot be detected, ligation of the internal iliac artery can be performed to reduce pulse pressure in the pudendal artery [9].

## 4 Conclusion

In summary, our report demonstrates that even large vulvar hematomas can be managed effectively with a simple incision procedure with bleeding ligation under local anesthesia if the hematoma is localized; confirming this localization is the most important step in vulvar hematoma management, and CT is the preferred imaging method.

## Consent

Written informed consent was obtained from the patient for publication of this case report and accompanying images.

A copy of the written consent is available for review by the Editor-in-Chief of this journal upon request.

## Provenance and peer review

Not commissioned, externally peer-reviewed.

## Ethical approval

Case reports do not require ethical approval by our institution.

## Funding

This study did not receive any funding.

## Guarantor

Brahmana Askandar Tjokroprawiro.

## Research registration number

Not applicable.

## CRediT authorship contribution statement

Sole author contributed to the manuscript preparation including data collection, literature review, drafting, writing and editing.

## Declaration of competing interest

The authors have no conflicts of interest to declare.
